# Supplementary material for: Investigating the Contribution of Major Drug-Metabolising Enzymes to Possum-Specific Fertility Control
Source: Int J Mol Sci. 2023 May 29;24(11):9424. doi: 10.3390/ijms24119424 (PMC10253456; doi:10.3390/ijms24119424)
Supplement: Supplementary file 1 [file ijms-24-09424-s001.zip › ijms-2381673-supplementary.pdf]

Supplementary Table S1. CYP3A catalytic activity in possum and mouse liver microsomes. (n=1).

Compounds	Species	Percent control catalytic activity at estimated IC_{50}	Percent control catalytic activity at estimated 2-fold IC_{50}
Ketoconazole*	Possum	76	69
	Mouse	78	53
Ritonavir*	Possum	85	76
	Mouse	97	85
Alizarin*	Possum	70	77
	Mouse	91	89
Galeterone*	Possum	76	80
	Mouse	88	86
Sulfaphenazole*	Possum	93	84
	Mouse	96	100
Gentiopicroside*	Possum	98	83
	Mouse	92	85
Chlorzoxazone*	Possum	83	83
	Mouse	89	107
Benzbromarone*	Possum	83	78
	Mouse	97	96
Terfenadine*	Possum	99	83
	Mouse	105	99
7-Hydroxyflavone*	Possum	81	78
	Mouse	97	97
Curdione*	Possum	92	103
	Mouse	143	134
WAY-234759*	Possum	81	82
	Mouse	100	103
WAY-325412*	Possum	90	77
	Mouse	97	94
WAY-325945*	Possum	74	56
	Mouse	89	90
WAY-657644*	Possum	95	80
	Mouse	91	97
Cobicistat*	Possum	115	97
	Mouse	83	55
WAY-324829*	Possum	102	89
	Mouse	97	97
Naringenin*	Possum	84	-
	Mouse	71	-
Tak-700	Possum	154	103
	Mouse	69	78
Naringin Dihydrochalcone*	Possum	111	87
	Mouse	92	96
Fluconazole*	Possum	107	107
	Mouse	70	80

**Voriconazole**	Possum	99	83
	Mouse	82	78
**Deferasirox**	Possum	93	-
	Mouse	87	-
**Ozagrel HCl**	Possum	121	95
	Mouse	92	85
**Avasimibe**	Possum	151	116
	Mouse	91	92
**Abiraterone Acetate**	Possum	128	110
	Mouse	85	89
**Cyclosporin A***	Possum	109	113
	Mouse	103	71
**Diosmetin**	Possum	84	-
	Mouse	15	-
**Ozagrel**	Possum	97	109
	Mouse	93	92
**Clarithromycin***	Possum	90	117
	Mouse	89	81
**Uniconazole**	Possum	82	84
	Mouse	67	40
**Cedrol**	Possum	107	99
	Mouse	90	62
**Diallyl sulfide**	Possum	103	96
	Mouse	72	84
**Polygalaxanthone III**	Possum	102	108
	Mouse	92	94
**Ginsenoside F1**	Possum	120	-
	Mouse	82	-
**2'-Hydroxyacetophenone***	Possum	108	105
	Mouse	112	80
**Itraconazole**	Possum	141	120
	Mouse	91	91
**CDD3505**	Possum	129	145
	Mouse	88	89
**Acetylshikonin**	Possum	128	112
	Mouse	100	93
**Posaconazole**	Possum	93	92
	Mouse	101	74
**Thiabendazole**	Possum	94	92
	Mouse	85	95
**Methoxsalen**	Possum	109	96
	Mouse	95	97
**Memantine HCl**	Possum	117	109
	Mouse	104	98
**Pioglitazone HCl**	Possum	99	87
	Mouse	98	100
**Apigenin**	Possum	94	121
	Mouse	116	106
**Baicalein**	Possum	102	112

	Mouse	114	99
**Piperine**	Possum	127	121
	Mouse	126	107
**Sodium Danshensu**	Possum	116	118
	Mouse	98	107
**PF-4981517**	Possum	98	108
	Mouse	101	110
**2,6-Dihydroxyanthraquinone**	Possum	97	102
	Mouse	106	128
**Stiripentol**	Possum	109	105
	Mouse	119	102
**Galangin**	Possum	115	103
	Mouse	131	123
**Rhapontigenin***	Possum	103	103
	Mouse	116	157
**Pimpinellin**	Possum	133	-
	Mouse	171	-
**Isosilybin**	Possum	73	-
	Mouse	71	-
**Dauricine**	Possum	108	117
	Mouse	113	112
**Bergaptol**	Possum	129	-
	Mouse	121	-
**TMS**	Possum	105	125
	Mouse	126	140
**Danshensu**	Possum	113	-
	Mouse	97	-

Results show percent control activity of compounds screened at estimated IC_{50} and 2-fold IC_{50} in possum and mouse liver microsomes (n=1).

(*) Indicates that these compounds (which are likely to further inhibit possum CYP3A catalytic activities) were further screened (n=3).

Supplementary Table S2. CYP3A catalytic activities of selected compounds in mouse, possum, avian, and human liver microsomes.

Compounds	Species	Screened concentration (μM)	Solvent control	Catalytic activity at IC_{50} +	% of control	Catalytic activity at 2-fold IC_{50} +	% of control
**Ketoconazole**	**Mouse**	**2.5 and 5**	0.58±0.02	0.27±0.04*	**46***	0.23±0.03*	**40***
	**Possum**		0.35±0.03	0.29±0.04	**83***	0.26±0.04	**73***
	**Avian**		0.31±0.04	0.25±0.04	**82***	0.22±0.04	**71***
	**Human**		0.54	0.43	**80**	0.43	**80**
**Fluconazole**	**Mouse**	**13.1 and 26.2**	0.46±0.04	0.42±0.04	**90**	0.42±0.05	**90**
	**Possum**		0.38±0.02	0.36±0.02	**96**	0.35±0.02	**92**
	**Avian**		0.33±0.03	0.32±0.05	**94**	0.33±0.02	**101**
	**Human**		0.66	0.61	**91**	0.60	**91**
**Ritonavir**	**Mouse**	**0.014 and 0.028**	0.62±0.05	0.58±0.04	**95**	0.52±0.04	**84***
	**Possum**		0.37±0.04	0.32±0.03	**87***	0.29±0.02	**81***
	**Avian**		0.30±0.02	0.27±0.02	**92***	0.26±0.01	**88***
	**Human**		0.42	0.40	**95***	0.40	**95***
**Cyclosporin A**	**Mouse**	**6 and 12**	0.49±.0.3	0.47±0.02	**98**	0.47±0.07	**96**
	**Possum**		0.33±0.02	0.35±0.01	**105**	0.38±0.05	**115**
**Naringenin**	**Mouse**	**1349 and 2698**	0.46±0.05	0.71±0.06*	**158***	1.09±0.04*	**240***
	**Possum**		0.32±0.02	0.32±0.01	**99**	0.56±0.02*	**175***
**Naringin Dihydrochalcone**#	**Mouse**	**1 and 5**	0.53±0.06	0.51±0.07	**97**	0.54±0.07	**102**
	**Possum**		0.36±0.02	0.38±0.03	**106**	0.33±0.02	**92**
**Clarithromycin**	**Mouse**	**1274 and 2548**	0.63±0.06	0.67±0.14	**104**	0.60±0.09	**95**
	**Possum**		0.42±0.03	0.39±0.04	**93**	0.43±0.01	**103**
	**Avian**		0.43±0.06	0.39±0.04	**91**	0.41±0.07	**94**
	**Human**		0.65	0.62	**95**	0.59	**91**
**Alizarin**#	**Mouse**	**1 and 5**	0.59±0.04	0.58±0.06	**98**	0.57±0.06	**96**
	**Possum**		0.33±0.03	0.27±0.00	**82**	0.27±0.01	**84**
	**Avian**		0.32±0.02	0.29±0.02	**90**	0.30±0.04	**94**
	**Human**		0.58	0.64	**110**	0.57	**98**
**Galeterone**#	**Mouse**	**1 and 5**	0.60±0.05	0.68±0.12	**112**	0.63±0.10	**105**
	**Possum**		0.38±0.03	0.35±0.04	**92**	0.32±0.02	**83**
	**Avian**		0.35±0.04	0.34±0.04	**99**	0.36±0.04	**105**
	**Human**		0.53	0.53	**99**	0.61	**114**
**Cobicistat**	**Mouse**	**30 and 60**	0.48±0.07	0.47±0.06	**80**	0.36±0.08	**68**
	**Possum**		0.41±0.06	0.36±0.04	**91**	0.30±0.02	**76**
	**Avian**		0.30±0.03	0.20±0.01	**70**	0.19±0.01	**63***
	**Human**		0.46	0.36	**78**	0.36	**78**

**Sulfaphenazole**	**Mouse**	**0.32 and 0.64**	0.63±0.01	0.67±0.05	**106**	0.68±0.06	**109**
	**Possum**		0.33±0.02	0.38±0.04	**115**	0.37±0.04	**111**
	**Avian**		0.30±0.03	0.30±0.04	**99**	0.33±0.06	**110**
	**Human**		0.45	0.45	**101**	0.47	**105**
**Gentiopicroside#**	**Mouse**	**1 and 5**	0.58±0.12	0.59±0.13	**101**	0.56±0.12	**97**
	**Possum**		0.35±0.01	0.32±0.01	**91**	0.29±0.02	**82**
	**Avian**		0.28±0.02	0.31±0.04	**111**	0.32±0.03	**114**
	**Human**		0.44	0.55	**125**	0.54	**122**
**Chlorzoxazone#**	**Mouse**	**1 and 5**	0.55±0.05	0.50±0.03	**91**	0.58±0.05	**107**
	**Possum**		0.28±0.05	0.23±0.04	**81**	0.23±0.04	**83**
	**Avian**		0.36±0.02	0.33±0.05	**91**	0.30±0.03	**84**
	**Human**		0.41	0.42	**102**	0.39	**95**
**Benzbromarone#**	**Mouse**	**1 and 5**	0.59±0.04	0.58±0.07	**97**	0.57±0.08	**97**
	**Possum**		0.33±0.03	0.28±0.02	**85***	0.26±0.02	**80***
	**Avian**		0.32±0.00	0.35±0.01	**108**	0.34±0.01	**104**
	**Human**		0.33	0.36	**109**	0.37	**113**
**Terfenadine#**	**Mouse**	**1 and 5**	0.60±0.06	0.56±0.02	**94**	0.49±0.08	**83**
	**Possum**		0.33±0.03	0.29±0.05	**88**	0.26±0.03	**79***
	**Avian**		0.36±0.04	0.35±0.04	**99**	0.34±0.04	**96**
	**Human**		0.40	0.40	**101**	0.39	**98**
**7-Hydroxyflavone**	**Mouse**	**100 and 200**	0.44±0.02	0.38±0.03	**87**	0.40±0.04	**90**
	**Possum**		0.39±0.05	0.32±0.04	**83**	0.38±0.08	**95**
	**Avian**		0.40±0.06	0.40±0.07	**99**	0.42±0.07	**103**
	**Human**		0.65	0.63	**97**	0.61	**95**
**Rhapontigenin**	**Mouse**	**0.4 and 0.8**	0.36±0.03	0.42±0.01	**116**	0.52±0.05*	**144***
	**Possum**		0.33±0.04	0.34±0.04	**106**	0.40±0.03	**124***
	**Avian**		0.30±0.03	0.29±0.03	**96**	0.30±0.03	**101**
	**Human**		0.48	0.51	**106**	0.53	**110**
**Curdione**	**Mouse**	**16.86 and 33.72**	0.41±0.03	0.46±0.03	**114**	0.38±0.05	**94**
	**Possum**		0.38±0.02	0.34±0.02	**91**	0.37±0.03	**99**
	**Avian**		0.42±0.06	0.41±0.06	**97**	0.41±0.04	**99**
**2'-Hydroxyacetophenone#**	**Mouse**	**1 and 5**	0.46±0.05	0.59±0.06	**129***	0.35±0.05	**75***
	**Possum**		0.28±0.05	0.33±0.44	**120***	0.29±0.05	**102**
	**Avian**		0.36±0.04	0.36±0.05	**101**	0.36±0.05	**102**
**WAY-234759#**	**Mouse**	**1 and 5**	0.56±0.10	0.53±0.10	**95**	0.60±0.13	**105**
	**Possum**		0.33±0.06	0.30±0.04	**92**	0.31±0.03	**97**
	**Avian**		0.37±0.03	0.36±0.04	**96**	0.38±0.04	**101**
	**Human**		0.44	0.45	**103**	0.49	**112**

**WAY-324829**#	**Mouse**	**1 and 5**	0.64±0.01	0.66±0.03	**104**	0.66±0.02	**103**
	**Possum**		0.37±0.03	0.39±0.03	**105**	0.35±0.02	**94**
	**Avian**		0.37±0.03	0.36±0.05	**95**	0.36±0.04	**97**
	**Human**		0.44	0.42	**97**	0.41	**94**
**WAY-325412**#	**Mouse**	**1 and 5**	0.59±0.06	0.60±0.06	**101**	0.55±0.07	**92**
	**Possum**		0.31±0.06	0.30±0.04	**100**	0.25±0.04	**83***
	**Avian**		0.37±0.03	0.29±0.02	**78***	0.34±0.01	**91**
	**Human**		0.44	0.40	**90**	0.46	**103**
**WAY-325945**#	**Mouse**	**1 and 5**	0.62±0.03	0.54±0.04	**89**	0.42±0.09	**67***
	**Possum**		0.33±0.05	0.26±0.03	**81**	0.21±0.02	**65***
	**Avian**		0.37±0.03	0.34±0.06	**90**	0.31±0.04	**82**
	**Human**		0.44	0.41	**93**	0.42	**94**
**WAY-657644**#	**Mouse**	**1 and 5**	0.51±0.01	0.49±0.01	**95**	0.45±0.02	**88***
	**Possum**		0.29±0.04	0.26±0.05	**86***	0.24±0.03	**81***
	**Avian**		0.36±0.04	0.32±0.03	**91**	0.30±0.03	**85***
	**Human**		0.44	0.43	**97**	0.45	**101**

* Significantly different compared to their respective controls (P<0.05).

+ Catalytic activities expressed in nmol/mg/min.

# No reported IC_{50} values in CYP3A enzymes and therefore screened at 1 and 5 μM concentrations.

Supplementary Table S3. Selected compounds screened for *p*-nitrophenol inhibitory activities in 4 species at estimated IC_{50} and 2-fold IC_{50} values.

Compounds	Species	Screened concentration (μM)	Solvent control⁺	UGT2B4 catalytic activity at IC_{50} ⁺	% of control	UGT2B4 catalytic activity at 2-fold IC_{50} ⁺	% of control
**Ketoconazole**	**Mouse**	**50 and 100**	2.94±0.11	2.86±0.12	**87**	3.31±0.09	**113**
	**Possum**		3.79±0.13	3.17±0.21*	**117***	2.72±0.11*	**72***
	**Avian**		0.46±0.16	0.35±0.12	**79***	0.55±0.18	**123***
	**Human**		3.80	3.82	**103**	3.72	**98**
**Fluconazole**	**Mouse**	**2. 5 mM and 5 mM**	2.52±0.25	2.01±0.09	**81**	1.76±0.11*	**78**
	**Possum**		3.76±0.22	3.50±0.25	**93**	2.89±0.15*	**74**
	**Avian**		0.31±0.15	0.41±0.15	**224**	0.33±0.13	**173**
	**Human**		3.17	3.33	**112**	2.98	**94**
**Ritonavir**	**Mouse**	**50 and 100**	1.84±0.13	2.40±0.28	**130***	1.77±0.13	**96**
	**Possum**		2.95±0.19	2.91±0.03	**100**	2.70±0.17	**91**
	**Avian**		0.35±0.08	0.13±0.04	**37***	0.42±0.09	**123***
	**Human**		3.17	3.33	**117**	2.86	**90**
**Baicalein**	**Mouse**	**2.4 and 4.8**	3.25±0.09	3.10±0.08	**96**	3.08±0.04	**95**
	**Possum**		3.69±0.07	3.86±0.20	**105**	3.91±0.20	**106**
	**Avian**		0.37±0.06	0.21±0.05	**56***	0.17±0.06	**44***
	**Human**		3.94	3.81	**97**	3.95	**100**
**Cyclosporin A**	**Mouse**	**30 and 60**	2.04±0.09	2.01±0.06	**99**	1.62±0.12*	**80***
	**Possum**		2.21±0.06	3.08±0.15*	**139***	2.48±0.11	**112**
	**Avian**		0.57±0.10	0.61±0.09	**109**	0.50±0.07	**90**
**Piperine**	**Mouse**	**20 and 40**	2.74±0.11	3.06±0.20	**113**	2.70±0.07	**99**
	**Possum**		4.18±0.22	4.28±0.14	**103**	4.29±0.17	**103**
	**Avian**		0.66±0.11	0.59±0.18	**84**	0.55±0.06	**86**
	**Human**		3.93	3.70	**94**	3.62	**92**
**Clarithromycin**	**Mouse**	**100 and 200**	1.98±0.15	2.35±0.06	**120**	1.77±0.12	**90**
	**Possum**		3.65±0.26	3.93±0.24	**108**	3.51±0.28	**96**
	**Avian**		0.37±0.12	0.41±0.13	**110**	0.20±0.08	**48***
**7-hydroxyflavone**	**Mouse**	**10 and 20**	2.52±0.15	2.53±0.22	**100**	2.55±0.18	**101**
	**Possum**		3.90±0.02	3.91±0.02	**100**	3.92±0.02	**101**
	**Avian**		0.41±0.13	0.58±0.14	**150***	0.38±0.12	**92**
**Cobicistat**	**Mouse**	**55 and 110**	2.50±0.02	2.60±0.10	**104**	2.56±0.04	**102**
	**Possum**		3.63±0.16	3.55±0.28	**98**	3.24±0.21	**89**
	**Avian**		0.48±0.16	0.21±0.10	**33***	0.21±0.11	**55***
	**Human**		4.03	3.96	**110**	3.61	**90**

**Sulfaphenazole**	**Mouse**	**300 and 600**	2.43±0.05	2.51±0.11	**103**	2.27±0.02	**94**
	**Possum**		3.64±0.11	3.80±0.16	**105**	3.59±0.23	**99**
	**Avian**		0.74±0.12	0.56±0.13	**74***	0.57±0.14	**74***
	**Human**		3.47	3.55	**102**	3.38	**98**
**Chlorzoxazone**	**Mouse**	**300 and 600**	3.56±0.50	3.53±0.59	**98**	3.62±0.51	**102**
	**Possum**		3.84±0.54	3.59±0.78	**91**	3.85±0.51	**100**
	**Avian**		0.57±0.10	0.55±0.09	**96**	0.59±0.10	**103**
**Terfenadine**	**Mouse**	**20 and 40**	2.10±0.16	2.21±0.13	**106**	2.20±0.14	**105**
	**Possum**		3.28±0.15	3.60±0.07	**110**	3.51±0.09	**107**
	**Avian**		0.42±0.08	0.37±0.11	**84**	0.25±0.03	**62***
	**Human**		3.01	3.43	**114**	3.20	**106**
**Isosilybin**	**Mouse**	**100 and 200**	1.93±0.17	1.84±0.12	**96**	1.55±0.19	**80**
	**Possum**		3.42±0.08	2.67±0.11*	**78***	2.21±0.17*	**65***
	**Avian**		0.45±0.14	0.45±0.14	**101**	0.28±0.11	**67***
	**Human**		3.17	2.95	**93**	3.15	**107**
**Cedrol**	**Mouse**	**0.63 and 1.26**	3.07±0.17	2.57±0.05	**84**	2.57±0.16	**84**
	**Possum**		4.14±0.08	3.86±0.06	**93**	3.78±0.13	**92**
	**Avian**		0.63±0.16	0.63±0.20	**96**	0.56±0.13	**91**
	**Human**		3.92	3.85	**98**	3.85	**98**
**Ginsenoside F1**	**Mouse**	**50 and 100**	2.83±0.29	3.63±0.15*	**132**	4.36±0.01*	**157***
	**Possum**		3.82±0.24	3.80±0.08	**100**	4.14±0.18	**109**
	**Avian**		0.64±0.18	0.66±0.14	**110**	0.66±0.14	**99**
	**Human**		4.03	4.03	**101**	4.01	**100**
**Itraconazole**	**Mouse**	**100 and 200**	1.90±0.21	2.36±0.30	**125***	1.99±0.24	**106**
	**Possum**		3.00±0.11	3.07±0.14	**102**	3.06±0.25	**102**
	**Avian**		0.38±0.06	0.46±0.09	**118**	0.35±0.05	**94**
	**Human**		3.17	2.66	**95**	2.80	**88**
**Acetylshikonin**	**Mouse**	**58 and 116**	3.09±0.11	2.87±0.17	**93**	2.92±0.22	**94**
	**Possum**		3.68±0.23	3.54±0.20	**96**	3.70±0.18	**101**
	**Avian**		0.64±0.11	0.57±0.13	**87**	0.53±0.08	**84***
	**Human**		3.70	3.62	**98**	3.70	**100**

* Significantly different compared to their respective control (P<0.05).

+ Activities are expressed as μmol/mg/min.
